# Limited acclimation in leaf anatomy to experimental drought in tropical rainforest trees

**DOI:** 10.1093/treephys/tpw078

**Published:** 2016-12-19

**Authors:** Oliver Binks, Patrick Meir, Lucy Rowland, Antonio Carlos Lola da Costa, Steel Silva Vasconcelos, Alex Antonio Ribeiro de Oliveira, Leandro Ferreira, Maurizio Mencuccini

**Affiliations:** 1 School of Geosciences, The Crew Building, The King's Buildings, University of Edinburgh, Edinburgh, EH9 3JN, UK; 2 Research School of Biology, Australian National University, Canberra, ACT 2601, Australia; 3 College of Life and Environmental Sciences, University of Exeter, Exeter, EX4 4QD, UK; 4 Centro de Geosciências, Universidade Federal do Pará, Belém 66075-110, Brasil; 5 EMBRAPA Amazônia Oriental, Belém 66095-903, Brasil; 6 Museu Paraense Emílio Goeldi, Belém 66077-830, Brasil; 7 ICREA at CREAF, 08193Cerdanyola del Vallés, Spain

**Keywords:** Amazon, anatomical plasticity, gas exchange, leaf physiology, through-fall exclusion, water relations, water stress

## Abstract

Dry periods are predicted to become more frequent and severe in the future in some parts of the tropics, including Amazonia, potentially causing reduced productivity, higher tree mortality and increased emissions of stored carbon. Using a long-term (12 year) through-fall exclusion (TFE) experiment in the tropics, we test the hypothesis that trees produce leaves adapted to cope with higher levels of water stress, by examining the following leaf characteristics: area, thickness, leaf mass per area, vein density, stomatal density, the thickness of palisade mesophyll, spongy mesophyll and both of the epidermal layers, internal cavity volume and the average cell sizes of the palisade and spongy mesophyll. We also test whether differences in leaf anatomy are consistent with observed differential drought-induced mortality responses among taxa, and look for relationships between leaf anatomy, and leaf water relations and gas exchange parameters. Our data show that trees do not produce leaves that are more xeromorphic in response to 12 years of soil moisture deficit. However, the drought treatment did result in increases in the thickness of the adaxial epidermis (TFE: 20.5 ± 1.5 µm, control: 16.7 ± 1.0 µm) and the internal cavity volume (TFE: 2.43 ± 0.50 mm^3^ cm^−2^, control: 1.77 ± 0.30 mm^3 ^cm^−2^). No consistent differences were detected between drought-resistant and drought-sensitive taxa, although interactions occurred between drought-sensitivity status and drought treatment for the palisade mesophyll thickness (*P* = 0.034) and the cavity volume of the leaves (*P* = 0.025). The limited response to water deficit probably reflects a tight co-ordination between leaf morphology, water relations and photosynthetic properties. This suggests that there is little plasticity in these aspects of plant anatomy in these taxa, and that phenotypic plasticity in leaf traits may not facilitate the acclimation of Amazonian trees to the predicted future reductions in dry season water availability.

## Introduction

A key issue in the prediction of future climate change is understanding how forests, significant stores of carbon (Pan et al. 2011, Grace et al. 2014), will respond to current and future changes in temperature and water availability (Bonan 2008). Tree mortality has reportedly increased in response to episodic severe drought (Breshears et al. 2005, Allen et al. 2010), including in the tropics (Nakagawa et al. 2000, Meir and Grace 2005, Phillips et al. 2009, Brienen et al. 2015), and understanding the physiology underlying drought-induced mortality is essential for estimating forest sensitivity to drought (Christensen et al. 2013, Allen et al. 2015, Meir et al. 2015*b*). Although spatial variation in predicted rainfall patterns is substantial, current consensus suggests that precipitation extremes in the tropics, and especially in Amazonia, are likely to become more frequent, with extended dry seasons of particular note (Christensen et al. 2013, Fu et al. 2013, Reichstein et al. 2013, Boisier et al. 2015). These changed conditions will exert a selection pressure affecting the next generation of trees, but the persistence of the current generation depends on their capacity for acclimation or resilience in the face of climate change. Investigating the capacity of trees to cope with drought in tropical forests is consequently of paramount importance in estimating the magnitude of biosphere–atmosphere feedbacks.

Species differ in their ability to cope with water stress (da Costa et al. 2010, Bartlett et al. 2012, Choat et al. 2012, Meir et al. 2015*a*, Rowland et al. 2015*b*) and establishing exactly what traits account for this differential susceptibility is complex, particularly in species-diverse communities. Globally, there have been reports of drought-induced tree mortality with the implicated cause being either carbon starvation or hydraulic failure, or a mixture of the two (McDowell 2011, Anderegg et al. 2012, Galiano et al. 2012, Anderegg et al. 2013, Hartmann et al. 2013, McDowell et al. 2013, Brodribb and McAdam 2015, Gleason et al. 2015). For the tropics, however, there have been few studies, with initial suggestions hinting at a role for progressive carbon starvation (Metcalfe et al. 2010) superseded by more recent evidence pointing towards hydraulic deterioration as a principal trigger for drought-induced mortality (Rowland et al. 2015*a*). Water stress in plants is commonly represented by the percent loss of hydraulic conductivity (PLC) of the xylem, whereby P50, the leaf or branch tissue water potential at 50% PLC, is used as a metric of the drought resistance of a plant or species, with higher (less negative) P50 indicating less resistance to the loss of conductivity through embolism. Attempts have been made to map P50 onto xylem anatomy, where wood density, conduit diameter and conduit wall thickness have been found to be weakly predictive of cavitation resistance (Hacke et al. 2001, Hajek et al. 2014, Gleason et al. 2015). Certain characteristics of leaves have also been shown to be associated with drought resistance in plants, e.g., turgor loss point (Bartlett et al. 2012) and the elastic modulus (Bowman and Roberts 1985), but the mechanistic relationships between leaf anatomy and drought resistance remain poorly understood.

Distinct morphological characteristics of leaves occur in environments of especially low or high water availability, and are termed xeromorphic and hygromorphic, respectively. Xeromorphic leaves tend to be small in area, with a multi-layered epidermis, thick cuticle, compactly arranged mesophyll with little air space, high stomatal density and high vein density (Maximov 1929, Cutler et al. 1977). By contrast, hygromorphic leaves tend to show the opposite features (Schimper 1903, Roth 1985). In certain species spanning gradients of rainfall, traits such as leaf area, thickness, specific leaf area, density, and stomatal morphology have been shown to vary with water availability (Geeske et al. 1994, Cunningham et al. 1999, Warren et al. 2005, McLean et al. 2014). Drought experiments have also shown reduced cell size in the mesophyll and epidermis, and increases in cell wall thickness, stomatal density, vein density and cuticle thickness in droughted vs non-droughted plants (Maximov 1929, Morton and Watson 1948, Shields 1950, Cutler et al. 1977).

Most of the experimental research on the adaptations of leaf anatomy to water stress was conducted over half a century ago on mesophytic crop plants (Maximov 1929, Morton and Watson 1948, Shields 1950, Cutler et al. 1977), but its relevance is current in the context of predicted changes in the hydrological regimes of tropical rainforests. Moreover, recent work has attempted to improve the mechanistic understanding of water movement from veins to stomata in leaves, by modelling the hydraulic pathway through cells, cell walls and as vapour through the internal airspaces (Rockwell et al. 2014*a*, 2014*b*, Buckley 2015, Buckley et al. 2015). Therefore, understanding the plastic response of leaves at the tissue level, may well be informative of the influence of cell structure on the hydraulic pathway, and how this affects water use at the leaf level. Thus, the question arises: can trees respond to long-term reductions in water availability by producing leaves that exhibit more xeromorphic characteristics? Such a capacity for acclimation could confer a significant advantage for long-lived canopy tree species (Nicotra et al. 2010), and could be important for determining the sensitivity of a forest to drought and the difference in drought sensitivity among species. Existing studies addressing this question in natural communities are observational (Geeske et al. 1994, Cunningham et al. 1999, McLean et al. 2014), thus, here we test the plasticity of leaf morphology experimentally, in tropical rainforest trees.

This study uses a long-term (>12 years) through-fall exclusion (TFE) experiment in the lowland Amazon Rainforest (da Costa et al. 2010, Meir et al. 2015*b*) to address the following questions: (i) do trees respond to long-term imposed soil moisture deficit through changes in leaf structure or anatomy? and (ii) do differences in anatomy, or anatomical plasticity, explain contrasts in drought sensitivity among taxa? We also examine any further associations between leaf anatomy, water relations and gas exchange traits and drought using multivariate analyses.

The expectation was that xeromorphic traits should be found particularly under conditions where leaves have to cope with drought stress. In particular, higher stomatal and vein density, smaller cell size in the spongy and palisade mesophyll, and lower cavity volume were expected to occur in the experimentally droughted forest and/or in individuals from drought-resistant genera. Additionally, to highlight potential links between anatomical and physiological properties of leaves, multivariate analyses were carried out combining leaf tissue properties with plant water relation traits and gas exchange parameters.

## Methods

### Study site

The field work was conducted in the Caxiuanã National Forest Reserve in the eastern Amazon Rainforest (1^o^43′S, 51^o^27′W). The field site is situated in lowland *terra firme* rainforest approximately 10–15 m above river level, has a mean temperature of ca. 25 °C, receives 2000–2500 mm of rainfall annually and has a dry season in which rainfall is <100 mm per month between June and November.

### Large-scale TFE experiment

The TFE experiment consists of a 1 ha plot from which approximately 50% of canopy through-fall has been excluded using plastic panels located 1–2 m above the ground since 2002. A 1 ha control plot, <50 m from the TFE, which has received normal rainfall, was also studied. The plots are divided into 10 m × 10 m subplots, of which the outer-most subplots were excluded from the study to account for possible edge effects on tree growth. Further details on experimental setup and results can be found in Fisher et al. (2007),Meir et al. (2009), Metcalfe et al. (2010), da Costa et al. (2010) and Rowland et al. (2015*b*).

### Study specimens and drought vulnerability status

All measurements (Table 1) were taken from six genera common to both the TFE and the control plot of which *Manilkara, Eschweilera* and *Pouteria* have been classified ‘drought sensitive’ and *Protium, Swartzia* and *Licania* as ‘drought resistant’, based on analysis of rates of drought-induced mortality (da Costa et al. 2010, Rowland et al. 2015*b*). These will be subsequently referred to as sensitive and resistant species. The sensitivity status of a genus is based on mortality in response to the imposed drought conditions. Where possible, a single species was used to represent a genus (*Pouteria anomala* (Pires) T.D. Penn.*, Manilkara bidentata* (A.DC.) A. Chev.*, Swartzia racemosa* (Benth.)), but more than one species was used where there were too few individuals in a species per plot. So *Eschweilera* is represented by the species *E. coriacea* (DC.) S.A. Mori*, E. grandiflora* (Aubl.) Sandwith, and *E. pedicellata* (Rich) S.A. Mori, *Licania* by *L. membranacea* (Sagot ex Laness) and *L. octandra* (Kuntze) and *Protium* by *P. tenuifolium* Engl. and *P. paniculatum* Engl. This approach was necessary to obtain sufficient numbers of each genus per plot to enable a comparison of drought sensitivity groups, i.e., in order that drought-sensitive and -resistant taxa were represented by three genera in both plots, and was used in two previous studies undertaken at the same site (Rowland et al. 2015*b*, Binks et al. 2016). In Binks et al., variance was consistently greater among genera than amongst individuals within genera. Similarly, in this study variance was also greater among, than within, genera in 13 out of 17 traits, the exceptions being leaf area, leaf mass per area and the proportional tissue thicknesses of the spongy mesophyll and abaxial epidermis (Table 2).

**Table 1.** Data transformations and final model structures used in analysis for the effect of treatment (T, control plot vs TFE) and drought-sensitivity status (S, sensitive or resistant) on tissue parameters, and gas exchange parameters used in PCA. The random effects were tree individual nested inside genera for all models with the exception of CV_prop_, for which tree individual was not used because of sample size limitations (see Table S1 available as Supplementary Data at *Tree Physiology* Online).

Leaf properties	Response variable	Symbol	Transformation	Model structure
Structural properties	Leaf area	A	log	T*S
	Leaf thickness	T	log	T*S
	Leaf mass per area	LMA	log	T*S
	Vein density	VD	*y*^2^	T*S
	Stomatal density	SD	–	T*S
Tissue properties	Spongy mesophyll thickness	SM	–	T*S
	Adaxial epidermis thickness	Ad	log	T
	Abaxial epidermis thickness	Ab	log	T*S
	Internal cavity volume	CV	–	T*S
	Proportional SM thickness	Sm_prop_	*y*^2^	T+S
	Proportional Ab thickness	Ab_prop_	log	T*S
	Proportional Ad thickness	Ad_prop_	log	T*S
	Proportional CV thickness	CV_prop_	√*y*	T*S
Cellular properties	SM cell volume	SM_cell_volume_	√*y*	T*S
	Pal cell volume	Pal_cell_volume_	log	T*S
Gas exchange parameters	Rubisco carboxylation	*V*_cmax_	–	PCA
	Electron transport	*J*_max_	–	PCA
	Dark respiration	*R*_dark_	–	PCA

**Table 2.** Variance accounted for by separate components in the mixed models and the conditional and marginal *r*^2^ of each model.

Variable	Variance (%)				*r*^2^_Conditional_	*r*^2^_Marginal_
	Fixed	Random		Residual		
		Gn	ID			
T	5.8	58.3	29.9	6.0	0.94	0.06
A	2.0	32.8	33.9	31.3	0.69	0.02
Ad	1.8	71.5	<0.1	26.7	0.73	0.02
SM	8.0	45.5	27.8	18.8	0.81	0.08
Pal	7.2	39.8	35.9	17.0	0.83	0.07
CV	9.0	55.2	29.9	5.9	0.94	0.09
Ab	4.2	45.1	24.5	26.1	0.74	0.04
Ad_prop_	16.7	63.1	7.2	13.0	0.87	0.17
Sm_prop_	19.0	26.2	33.0	21.8	0.78	0.19
Pal_prop_	11.8	33.1	28.5	26.6	0.73	0.12
CV_prop_	18.1	47.4	–	34.5	0.76	0.06
Ab_prop_	14.3	15.7	40.2	29.8	0.70	0.14
VD	1.9	69.3	6.7	22.0	0.78	0.02
SD	3.8	39.9	38.6	17.8	0.82	0.04
LMA	4.1	13.1	44.8	38.0	0.62	0.04
SM_cell_volume_	23.8	20.5	14.7	40.9	0.59	0.24
Pal_cell_volume_	20.3	49.0	10.1	20.6	0.79	0.20

For each of the variables, an attempt was made to measure at least two leaves per individual tree, and three individual trees per genus per plot which would have resulted in 18 leaves from nine individuals per drought sensitivity status, per plot. Unfortunately, it was not always possible to achieve this number of samples, due to difficulties obtaining suitable leaves and performing the analysis under field conditions. Moreover, peculiarities in some of the specimens made particular analyses difficult or impossible; e.g., leaves from the genus *Manilkara* were densely packed with sclereids, which obscured the vasculature and made accurate analysis of vein density impossible under the conditions available. Therefore, the minimum and mean number of leaves per genus, genus per plot and drought sensitivity status per plot are detailed in Table S1 (available as Supplementary Data at *Tree Physiology* Online) for each analysis. The minimum number of leaves per sensitivity status per plot, which is the analytical unit, ranged from 7 (relative internal cavity volume) to 93 (leaf area and leaf mass per area), with a median of 13.

All leaves were taken from branches sampled at the top of dominant trees only and exposed to direct sunlight for a portion of the day; shaded leaves were not used in this analysis. After excision, the branches were transported in buckets of water back to the field station, and were recut under water filtered to 0.2 µm and allowed to rehydrate overnight. The leaves used in all analyses were fully expanded and mature. Experimental procedures were carried out in May and June in 2014 with the exception of pressure–volume curves, which were also measured in October and November of 2013.

### Lamina anatomy

Small squares of leaf of area >0.5 cm^2^ situated midway between the leaf tip and base, and midrib and margin of the leaf were sectioned using a hand-held microtome. The sections were photographed at a magnification of ×40 in cases where this enabled the whole depth of the leaf to be observed in one image, and at ×10 for thicker leaves. Images were analysed in ImageJ to obtain thickness values in microns for the adaxial (Ad) and abaxial (Ab) epidermis layers, the palisade mesophyll (Pal) and the spongy mesophyll (SM). Accurate measurements of cuticle thickness were not possible as they were of a similar magnitude to the standard error on the measurement of epidermal thickness (Table 3). Therefore, cuticle thickness is included in the values for epidermis thickness. The thickness of each tissue layer was also presented as a percentage of total leaf thickness (*T*, µm) and indicated by the subscript ‘prop’, e.g., Ad_prop_.

**Table 3.** Mean value by genus of each of the leaf tissue parameters ± 1 standard error.

	Drought sensitive			Drought resistant		
	*Eschweilera*	*Manilkara*	*Pouteria*	*Licania*	*Protium*	*Swartzia*
*T* (µm)	164.7 ± 4.7	247.2 ± 15.9	168.9 ± 6.7	165.5 ± 27.7	123.5 ± 9.9	248.4 ± 8.4
*A* (cm^2^)	69.5 ± 4	29.9 ± 1.5	43.4 ± 1.7	40.8 ± 1.8	47.1 ± 2.1	39.4 ± 2
Pal (µm)	35.9 ± 1.3	66.7 ± 8.1	60.6 ± 6.5	56.8 ± 6.5	42.8 ± 3.2	66.8 ± 3.8
SM (µm)	100.6 ± 4.7	150.5 ± 9.1	82.9 ± 7.7	71.9 ± 17.7	59.2 ± 5.4	139.9 ± 5.5
Ad (µm)	15.9 ± 0.8	13.1 ± 1.5	15.6 ± 0.8	34.3 ± 1.8	12.4 ± 1.5	19.9 ± 1.1
Ab (µm)	12.3 ± 0.6	15.4 ± 1.9	13.1 ± 0.7	15.6 ± 2.3	9.2 ± 0.9	22.5 ± 2
CV (mm^3^ cm^-2^)	2.1 ± 0.3	3.3 ± 0.2	0.1 ± 0	3.1 ± 0.7	0.3 ± 0	2.5 ± 0.4
VD (mm mm^-2^)	8.4 ± 0.3	–	9.6 ± 0.2	11.4 ± 0.6	11.5 ± 0.3	6.3 ± 0.2
SD (mm^-1^)	409.7 ± 12.2	317.3 ± 20.6	350.1 ± 28.8	306.1 ± 32.3	563.5 ± 12.7	301 ± 10.5
LMA (g m^-2^)	91.1 ± 1.7	125.8 ± 5.8	103.3 ± 3.6	116.6 ± 4.8	90.6 ± 1.6	115.4 ± 2.7
Pal_prop_ (%)	22.2 ± 1.1	26.3 ± 2.1	35.9 ± 3.7	37.1 ± 3.4	34.9 ± 1.1	26.4 ± 1.3
SM_prop_ (%)	60.6 ± 1.6	63.7 ± 2.2	46.8 ± 3.4	41.6 ± 4.3	47.7 ± 1.1	55.9 ± 2.1
Ab_prop_ (%)	7.6 ± 0.4	5.2 ± 0.5	7.9 ± 0.5	9.8 ± 0.8	7.5 ± 0.5	9.5 ± 1.1
Ad_prop_ (%)	9.6 ± 0.4	4.9 ± 0.6	9.4 ± 0.6	24.1 ± 2.8	10 ± 0.9	8.3 ± 0.5
SM_cell_volume_ (µm^3^)	3456 ± 421	2614 ± 224	2641 ± 295	884 ± 67	946 ± 193	2656 ± 348
Pal_cell_volume_ (µm^3^)	1220 ± 106	3695 ± 263	4371 ± 440	823 ± 109	1797 ± 204	2046 ± 683

Mean cell volume for the palisade and spongy mesophyll was calculated by assuming that palisade cells were cylinders and spongy mesophyll cells were spheres. Mean values for the length and width of palisade cells, and the width of spongy mesophyll cells, were determined from five cells per leaf section, and averaged between two leaf sections per individual tree.

### Mesophyll cavity volume

Branches collected during the afternoon were covered and left to rehydrate overnight. Leaves were only used if adjacent leaves had a water potential higher than −0.2 MPa. Specimen leaves were then perfused with tap water filtered to 0.02 µm for >20 h at a pressure of 18 kPa. The cavity volume (CV) was determined by subtracting the fresh leaf mass from the perfused leaf mass expressed as mm^3^_air space_ cm^−2^_leaf surface_.

### Vein density

Small squares of leaf approximately 1 cm^2^ in size taken from midway between the tip and base, and midrib and margin of the leaf were cleared using 5% NaOH and briefly 10% NaClO when necessary to remove the last of the colour (Scoffoni et al. 2010). The cleared leaf sections were then placed in a 1% solution of toluidine blue for several seconds before being rinsed in water; this process was repeated until sufficient dye was judged to have infiltrated the sample and the veins were clearly visible. The samples were photographed using a Moticam 2 digital camera on a Motic B3 microscope (Motic, Canada). An objective lens of ×10 magnification was used for most images, but ×4 magnification was used where Student's *t*-test revealed no significant difference (*P* > 0.05) in the vein densities calculated from either magnification. Vein density (VD, mm mm^−2^) was derived by tracing and measuring vein length in a known area using ImageJ software (Schneider et al. 2012). Where there was a clear distinction in size between second and third order veins, only those in the third order and above were included in the analysis (where the midrib counts as the first order).

### Stomatal density

Dental impression gel was used to cover a minimum area of 2 cm^2^, situated midway between the tip and base, of the abaxial surface of four leaves per individual and the adaxial surface of one leaf per individual. Clear nail varnish was applied to the surface of the dental impressions, peeled off and photographed using a Leica DFC420 C camera mounted on a Leica DMLB 100 S transmission light microscope (Leica, Germany). Two photos were taken per leaf impression and stomatal density (SD, mm^−2^) was derived by counting the number of stomata in an area of 0.1–0.15 mm^3^ using ImageJ.

### Pressure volume analysis

Pressure–volume (PV) curves were carried out as per the bench-drying protocol described in Tyree and Hammel (1972). Briefly, leaves were taken from branches that had been rehydrated by allowing to stand overnight in a bucket of water filtered to 0.2 µm. The leaves were allowed to dehydrate over a period of 3–8 h, during which time water potential and mass were measured repeatedly using a Scholander pressure bomb (PMS Instruments Co., Corvalis, OR, USA) and mass balance accurate to 0.1 mg, respectively. After the final set of measurements, leaves were scanned to enable the determination of area using ImageJ software (Schneider et al. 2012) and then dried at 70 °C in an oven for >48 h to find dry mass. The parameters osmotic potential at full turgor ($\Psi_{\pi }^{o}$, MPa), turgor loss point ($\Psi_{\pi }^{\mathrm{tlp}}$, MPa), saturated water content (the ratio of water mass to leaf dry mass in a fully saturated leaf, SWC, g g^−1^), relative water content at *π*_tlp_ (RWC^tlp^, %), modulus of elasticity (*ε*, MPa) and hydraulic capacitance (*C*, mol MPa^−1^ m^−2^) were calculated as per Sack and Pasquet-Kok (2011). Differences in PV parameters across drought sensitivity status and plots are reported in a separate paper (Binks et al. 2016). Here, we present a correlation analysis including PV, leaf anatomy and gas exchange parameters in the Supplementary Data. The PV parameters were averaged across the wet and dry season for the correlation analysis as the magnitude of seasonal variation is less significant than the differences between species (Bartlett et al. 2014), and thus the benefit of doubling the sample size outweighed the cost of the slight increase in variance. Moreover, seasonal variation was only significant in SWC, RWC^tlp^ and *C*, but not in $\Psi_{\pi }^{o}$, $\Psi_{\pi }^{\mathrm{tlp}}$ or *ε* (Binks et al. 2016).

### Photosynthesis

For a detailed description of how the gas exchange parameters were measured, refer to Rowland et al. (2015*b*), in which these parameters were presented in the context of the experimental drought. Photosynthesis was measured on canopy top branches using LICOR 6400 portable photosynthesis systems (LI-COR, Lincoln, NE, USA). The parameters *V*_cmax_ (the maximum rate of Rubisco carboxylation) and *J*_max_ (the maximum rate of electron transport) were derived from A−C_i_ curves performed under saturating photosynthetically active radiation (PAR), and data were temperature corrected to 25 °C following Sharkey et al. (2007). To measure dark respiration (*R*_dark_) leaves were covered in tin foil for 30 min prior to, and during, gas exchange measurements, and these data were also temperature corrected to 25 °C according to Atkin and Tjoelker (2003).

### Predawn water potentials

Water potential was measured in three leaves per individual tree, between 5.30 and 7.00 in the morning at the end of the dry season (October) in 2013. Values were averaged for each individual tree.

### Analysis

The data were analysed using linear mixed effects models in the packages lmer and lmerTest in R (R Core Team 2015). Because the study was focused on finding treatment and drought sensitivity effects, and not the responses of individual genera, genus was designated as a random effect, and tree individual was nested inside genus, where there were >2 individuals per genus per plot. This statistical design removes the variance attributable to individuals within genera, and between genera, in order to selectively find the influence of the fixed effects, e.g., treatment and drought sensitivity status. Models (Table 1) were simplified by comparing their respective AIC. The distributions of all of the variables were checked for normality using the Shapiro–Wilk test and either log or power transformed depending on the starting distribution. The powers employed for transformation were determined using a Box–Cox transformation function in the MASS package in R (Venables 2002). The marginal and conditional *r*^2^ were calculated according to Nakagawa and Schielzeth (2013).

All of the anatomy variables were tested for correlations with the parameters derived from the PV analysis, predawn water potential (Ψ_PD_) and gas exchange (correlation matrix, see Table S2 available as Supplementary Data at *Tree Physiology* Online). The analysis was carried out using Pearson correlation analyses in the R package ‘psych’ (Revelle 2015) and all variables were transformed as per the mixed effects models (Table 1).

A principal component analysis (PCA) was performed on the thicknesses of leaf tissues, vein and stomatal density and the gas exchange-derived parameters *V*_cmax_, *J*_max_ and *R*_dark_ to highlight possible relationships between anatomy and photosynthesis. Absolute values of tissue thickness were used because of the relevance of distance, i.e., tissue thickness, to the molecular diffusion processes.

## Results

**Figure 1.**
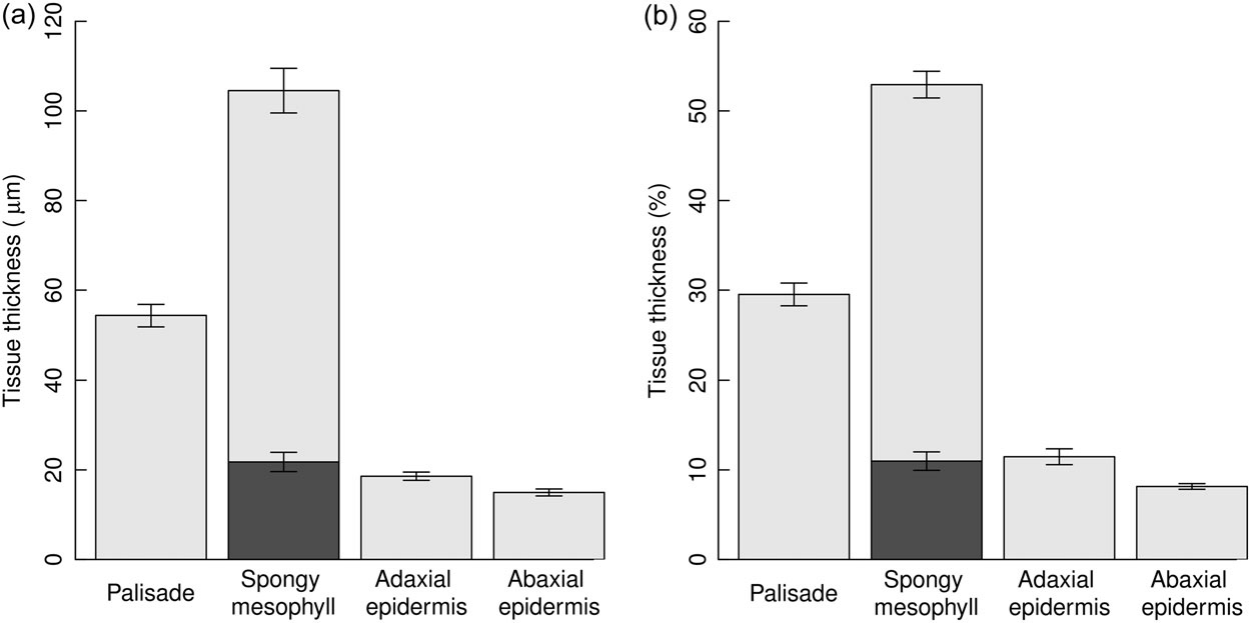
Mean absolute (a) and proportional (b) tissue thicknesses of studied taxa with standard error bars. Dark grey fraction of the spongy mesophyll bar represents the mean ‘thickness’ of the leaf cavity (total cavity volume/leaf area).

Leaf traits by genus are presented in Table 3. Leaf thickness varied from 78 to 370 µm with a combined mean and standard error of all genera and treatments of 187.6 ± 7.2 µm and a mean relative thickness with standard error of palisade, spongy mesophyll, abaxial and adaxial epidermis of 29.5 ± 1.3, 52.9 ± 1.49, 8.1 ± 0.3 and 11.5 ± 0.9%, respectively (Figure 1).

**Figure 2.**
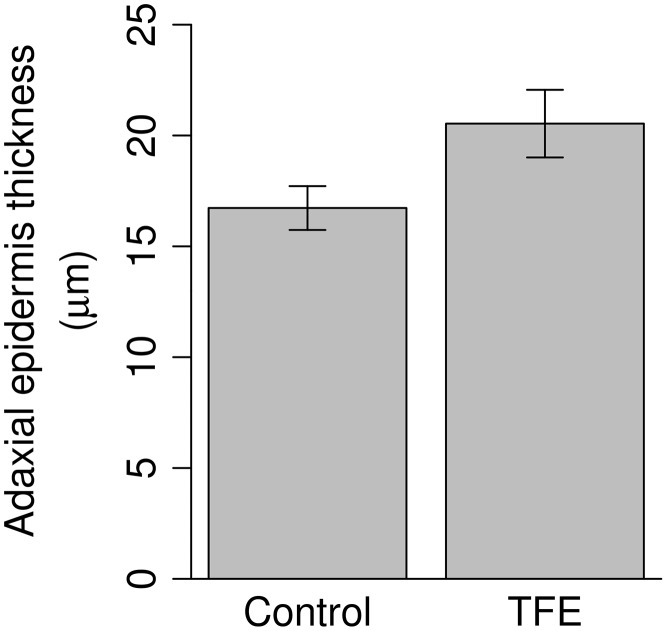
Treatment effect on the thickness of the adaxial epidermis of all studied taxa combined (*P* = 0.038).

**Figure 3.**
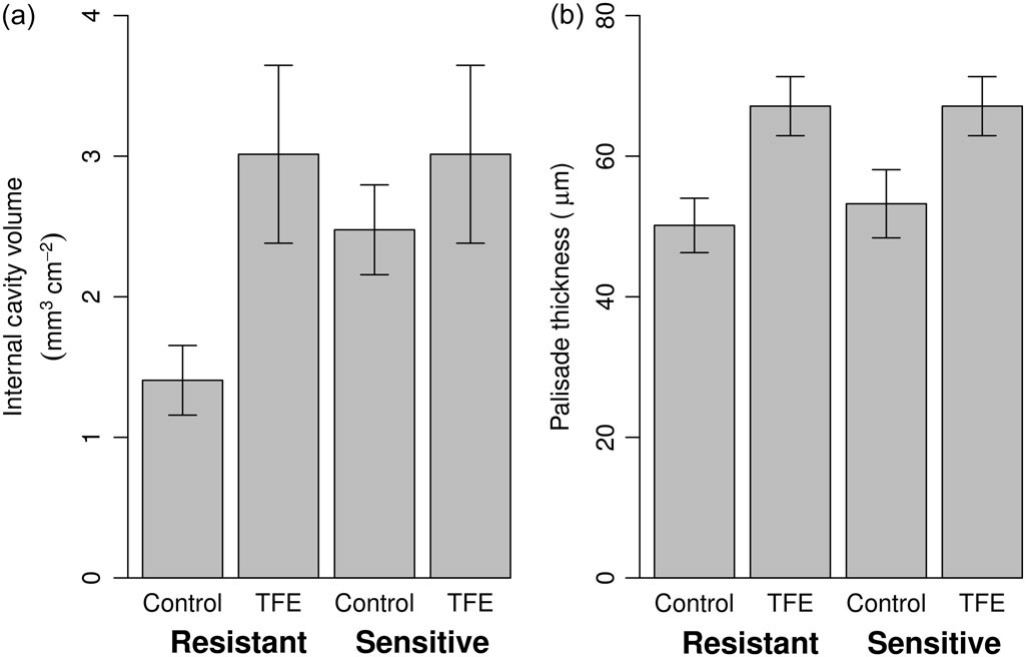
Interaction plots between drought sensitivity (resistant and sensitive taxa grouped by genus) and treatment for (a) internal cavity volume and (b) palisade thickness. Plots show means ± 1 standard error. The cavity volume shows significant treatment (*P* = 0.009) and interaction between treatment and drought sensitivity (*P* = 0.025) effects, while the palisade shows only significant interaction effects (*P* = 0.034).

Significant differences in response to treatment (*P* < 0.05) amongst all taxa combined were found for the absolute measure of cavity volume (TFE 2.43 ± 0.50 mm^3^ cm^−2^, control 1.77 ± 0.30 mm^3^ cm^−2^, Table 4) and the thickness of the adaxial epidermis (TFE 20.5 ± 1.5 µm, control 16.7 ± 1.0 µm, Table 4, Figure 2). Total leaf thickness (*P* = 0.070), palisade thickness (*P* = 0.086), the proportional cavity volume (*P* = 0.069) and LMA (leaf mass per area, *P* = 0.098) were found to be marginally significant (0.05 < *P* < 0.1). None of the measured variables showed significant differences between the two drought sensitivity classes (Table 4). However, CV and palisade thickness showed significant interactions between treatment and sensitivity status (*P* = 0.025 and 0.034, respectively), whereby the treatment effect was stronger (i.e., values were lower in the control plot) amongst the resistant compared to the sensitive genera (Figure 3).

**Table 4.** Probability values of the fixed effects included in the mixed models listed in Table 1, values in bold indicate a significant effect at *P* < 0.05. Factors with a dash were not included in the final model.

Variable	Treatment	Drought sensitivity	Interaction treat. × sens.
T	0.070	0.421	0.181
A	0.666	0.826	0.834
Ad	**0.038**	–	–
SM	0.543	0.488	0.757
Pal	0.086	0.618	**0.034**
CV	**0.009**	0.565	**0.025**
Ab	0.195	0.893	0.611
Ad_prop_	0.634	0.266	0.750
Sm_prop_	0.786	0.417	0.147
Pal_prop_	0.848	0.632	0.212
CV_prop_	0.069	0.832	0.054
Ab_prop_	0.972	0.173	0.832
VD	0.756	0.540	0.379
SD	0.797	0.638	0.470
LMA	0.098	0.591	0.278
SM_cell_volume_	0.914	0.083	0.896
Pal_cell_volume_	0.193	0.131	0.098

The variance accounted for the by the fixed effects (see Table 1 for model structures) varied from 1.8% for the adaxial epidermis to 23.8% for the spongy mesophyll cell volume with a mean of 10.1% over all of the variables (Table 2). In all but 4 out of 17 traits variance was higher among genera than within genera, averaging 42.7 and 27.1%, respectively, suggesting that the analysis of the effects was robust to pooling. Of the four traits where more variance occurs within a genus, leaf area, SM_prop_, Ab_prop_ and LMA, none were found to have significant treatment or drought sensitivity effects.

The correlation matrix of all the anatomical parameters, the PV parameters, the gas exchange parameters and predawn water potentials is given in Table S2 available as Supplementary Data at *Tree Physiology* Online. The thickness of the spongy mesophyll and adaxial epidermis correlated with more parameters than the other tissue layers, suggesting that they were tightly associated with other leaf traits, although they appeared to operate antagonistically, i.e., Ad_prop_ and SM_prop_ were negatively correlated with each other, and had opposite associations with the other leaf traits. Both Ad and Ad_prop_ were positively correlated with *ε* and Ψ_PD_. The photosynthesis parameters correlated positively with both palisade and spongy mesophyll thickness and negatively with vein and stomatal density, but did not correlate with the thickness of the epidermal layers.

**Figure 4.**
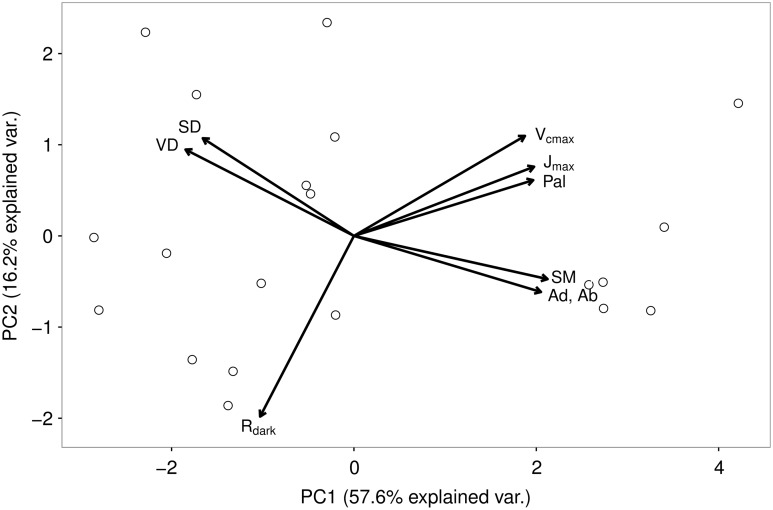
First two axes of the PCA showing distribution of tree individuals from all studied taxa based on absolute tissue thicknesses, vein and stomatal density, and the gas exchange parameters *V*_cmax_, *J*_max_ and *R*_dark_. Each point represents an individual tree. The spongy mesophyll (SM) is a measure of the tissue thickness (volume per area) without the cavity volume.

In the PCA, combining anatomical and gas exchange traits, the first and second axes explained 57.6 and 16.2% of the variance, respectively (Figure 4). Palisade thickness was grouped with *J*_max_ and *V*_cmax_ at high values of both axis 1 and 2, in the opposite quadrant to *R*_dark_. For the other parameters, there was a gradient of high vein and stomatal density, at low values of axis 1 and high values of axis 2, to high thickness of spongy mesophyll and epidermis in the opposite quadrant, which was orthogonal to the photosynthesis traits.

## Discussion

The results of this study reveal that little anatomical change occurred in response to the soil moisture deficit following 12 years of imposed through-fall exclusion. In addition, the drought sensitivity classification in these tropical forest trees, as determined by their increased mortality risk during drought stress, is not linked to specific leaf anatomy traits. We hypothesized that leaves would become more xeromorphic in character in response to the treatment, i.e., thicker, with smaller area, having lower internal cavity volume and higher stomatal and vein density. In fact, the only traits that did vary significantly in response to the treatment were the thickness of the adaxial epidermis (Figure 2) and the cavity volume, the second of which, contrary to expectation, increased in response to the imposed drought (Figure 3a).

### Drought sensitivity status

There were no significant differences in leaf anatomy based on sensitivity status, implying that other aspects of plant physiology determine sensitivity to water stress. However, the interaction between sensitivity status and treatment for cavity volume and palisade thickness indicates a possible link between drought sensitivity and plasticity in these traits. Values of cavity volume were similar among sensitive and resistant genera in the TFE, whereas the values of the resistant genera in the control plot were lower than the overall mean (Figure 3a). In other words, the acclimation response to the drought stress brought the value for CV amongst the resistant taxa in the TFE in-line with the values of the sensitive taxa in the TFE, bringing into question any drought-related benefit of plasticity in this trait. Therefore, there is no strong evidence in our dataset to suggest that the leaves of drought-resistant species are consistently different from those of drought-sensitive species.

Despite the strong general relationship between palisade thickness and photosynthesis (Chabot and Chabot 1977, Smith et al. 1998, Hanba et al. 2002, Catoni et al. 2015), and also observed in this data (see Table S2 available as Supplementary Data at *Tree Physiology* Online), earlier work by Rowland et al. (2015*b*) on the same experiment demonstrated no effect of plot or drought sensitivity on photosynthetic capacity or leaf nitrogen content. Therefore, the palisade is significantly thicker for the resistant species on the TFE (with a marginally significant difference between plots, Figure 3b, Table 4), but this has not resulted in higher photosynthetic capacity as determined by maximum rates of Rubisco carboxylation (*V*_cmax_) or electron transport (*J*_max_) (Rowland et al. 2015*b*). Moreover, the lack of significant change in *N* content suggests that Rubisco content (or Rubisco activation) has not changed considerably between treatments. These findings demonstrate that, at least in this case, the change measured in palisade thickness is not related to the maximum photosynthetic capacity. However, at least one other experiment has shown that the palisade mesophyll thickness increased in response to water deficit (Boughalleb and Hajlaoui 2011), suggesting that this trait may also influence water use within the leaf, or be influenced by water status during ontogeny.

### The drought effect

The cause of the higher CV in the drought plot (Figure 3a) is unknown as this is a feature of leaf morphology that has been explored little in the context of water stress, and the response of CV to long-term conditions of low VPD is not consistent among studies (Leuschner 2002, Aliniaeifard et al. 2014). We speculate that a higher internal cavity volume may reduce internal vapour pressure, both because of the effect of the larger cavity (greater distance between adjacent cell walls) and/or because of longer apoplastic path lengths resulting in lower local water potentials, with the consequence of potentially increasing photosynthetic water use efficiency (Mediavilla et al. 2001).

Several previous studies have linked thicker adaxial epidermis with drought resistance (Bacelar et al. 2004, Boughalleb and Hajlaoui 2011), although the causes for this remain uncertain. The results of this study show that the adaxial epidermis is thicker in the TFE and correlates positively with predawn water potential (see Table S2 available as Supplementary Data at *Tree Physiology* Online), while the results of a previous study, on the same taxa and individuals, showed that the thickness of the adaxial epidermis correlates negatively with osmotic potential at full turgor (Ψ_π_^0^) and turgor loss point (Ψ_π_^tlp^), and positively with the elastic modulus (*ε*) (Binks et al. 2016, see Table S2 available as Supplementary Data at *Tree Physiology* Online). If it is assumed that these correlations arose because turgor and/or osmotic properties in the Ad differ from those in other parts of the leaf, then the Ad would appear to be a particularly drought resistant tissue, e.g., low Ψ_π_^tlp^ and Ψ_π_^0^, and high *ε*. Thus, a thicker Ad would be linked to higher drought resistance which may explain why it is significantly thicker in the drought plot (Table 4, Figure 2).

### Water deficit and leaf expansion during ontogeny

The Lockhart equation explains the mechanical relationship between turgor pressure and the rate of cell expansion (*E*) in which *E = m*($\Psi_{P}$ − $\Psi_{P}^{\min}$) where $\Psi_{P}$ is turgor pressure, $\Psi_{P}^{\min}$ is the threshold turgor pressure below which growth does not occur, and *m* is the cell wall extensibility (Lockhart 1965). Leaves will, therefore, be smaller if their expansion phase occurs during periods of water stress (Shields 1950). Because vascular tissue and stomata are differentiated prior to expansion, reductions in leaf size effected during growth can be associated with increases in vein and stomatal density (Hsiao 1973, Schoch et al. 1980, Carins Murphy et al. 2014). However, no differences were detected in leaf area, cell size, vein or stomatal density in response to the drought treatment indicating that turgor pressure must not have dropped below the threshold minimum for long enough during the leaf expansion phase to influence these parameters in the mature leaves. All of the study species were evergreen but a partial leaf flush occurs at the beginning of the wet season (unpublished data) during which most leaves complete their growth. Thus, the wet season water supply on the TFE appears to be non-limiting to growth, which may be facilitated by reduced stomatal conductance to maintain adequately high cell turgor, and the reduction in leaf area index on the TFE (Metcalfe et al. 2010).

One factor that is interesting to consider in relation to the effects of experimental drought, is the potentially challenging aspect of separating mechanical effects of reduced turgor on growth, from the active expression of plastic traits that facilitate drought resistance, i.e., passive versus active plasticity (Valladares et al. 2007). In the context of leaves, this leads to the question of whether the traits that emerge as a consequence of expansion under sub-optimal turgor pressure, e.g., smaller area, higher vein and stomatal density and smaller mesophyll cells, actually provide an adaptive advantage for drier conditions, or are simply the product of drought stress during ontogeny. The similarity between leaf traits that emerge in response to experimental drought (Maximov 1929, Cutler et al. 1977), and those that differ along precipitation gradients (Geeske et al. 1994, Cunningham et al. 1999, Warren et al. 2005, McLean et al. 2014) presumably suggests the former: that these traits offer an adaptive advantage to coping with water stress.

### Correlations between leaf anatomy, gas exchange, predawn water potential and pressure volume traits

The correlation matrix revealed that several leaf tissues appear to be associated with many water relation and gas exchange traits (see Table S2 available as Supplementary Data at *Tree Physiology* Online), suggesting that these tissues are particularly representative of overall leaf physiology despite the limited treatment effects. These results must be interpreted with caution as the traits in this analysis are not independent; for example, leaf thickness is the sum of the thicknesses of all tissues layers, and similarly $\Psi_{\pi }^{\mathrm{tlp}}$ is a function of $\Psi_{\pi }^{o}$ and *ε*, so these traits inevitably correlate. Having said that, the proportional thickness of the adaxial epidermis and the absolute thickness of the spongy mesophyll correlated with a larger number of traits than the other tissues, and always in opposite directions (see Table S2 available as Supplementary Data at *Tree Physiology* Online). For example, SM correlates positively with $\Psi_{\pi }^{\mathrm{tlp}}$, SWC and RWC^tlp^ and negatively with SD, VD and $\Psi_{\mathrm{PD}}$, while Ad_prop_ shows the opposite relationships. Thus, leaves with thicker adaxial epidermis and thinner spongy mesophyll and, therefore, low $\Psi_{\pi }^{\mathrm{tlp}}$ and high $\Psi_{\mathrm{PD}}$, should be associated with greater drought resistance.

A high value of the elastic modulus is generally associated with drought resilience because it results in a greater change in Ψ for a given amount of water loss (Bowman and Roberts 1985), thus increasing the potential gradient and the capacity for rehydration. The correlation matrix reveals a positive relationship between *ε* and $\Psi_{\mathrm{PD}}$ supporting this theory, suggesting that high elastic modulus could be advantageous for nocturnal drought stress recovery in these taxa. In a previous study (Binks et al. 2016), *ε* was not found to vary significantly with drought sensitivity status but was significantly higher across all groups in the TFE than the control plot.

### Principal component analysis

Given the association of the palisade mesophyll with photosynthesis, it is not surprising that it is clustered with *J*_max_ and *V*_cmax_ in the PCA (Figure 4). However, it is surprising that stomatal and vein density are on a vector orthogonal to the photosynthesis traits, as in other studies they have been shown to correlate positively with photosynthesis (Brodribb et al. 2010, Walls 2011, Muller et al. 2014, Zhang et al. 2014), while, in this dataset, they correlate negatively (see Table S2 available as Supplementary Data at *Tree Physiology* Online). Acting in the opposite direction to VD and SD are the other leaf traits, SM, Ab and Ad, which may suggest that increases in the thickness of these tissues can compensate for the functions of otherwise higher vein and stomatal density. Past research analysing the movement of dye from leaf veins into the surrounding tissue indicates that the epidermal layers play a role in lateral water transport (Wylie 1943), which might explain the negative correlation between vein density and the epidermal layers (Figure 4, see Table S2 available as Supplementary Data at *Tree Physiology* Online).

### Acclimation to drought

The level of acclimation detected in this study was lower than expected suggesting a limitation to levels of plasticity in the measured traits in response to the experimental drought. The conditions that are thought to favour the selection for phenotypic plasticity are predictable variations in the environment within certain limits. If, in a given environment, a particular abiotic factor fluctuates very little, unpredictably, or to too extreme an extent for plastic responses to incur a significant increase in fitness, then phenotypic plasticity is not selected for in a population (Valladares et al. 2007). Thus, it is possible that the taxa in this study have limited capacity to acclimate to drought because of the historical stability or unpredictability in water availability. Other factors that may contribute to the limited response include the concept of ‘integrated phenotype’ where traits are so tightly interdependent that changes in one aspect of physiology impact, perhaps negatively, on other aspects (Gianoli 2001, Valladares et al. 2007), or the effects of resource limitation inhibiting a plastic response (Van Kleunen and Fischer 2005). Moreover, plants are rarely specialised to cope with more than one kind of abiotic stress (Niinemets and Valladares 2006), so perhaps the same is true of plasticity in certain traits, and the studied taxa may show higher levels of plasticity in response to, e.g., different levels of irradiance, which may be more advantageous to rainforest species.

Although the changes that were expected did not occur, e.g., higher stomatal and vein density, smaller cell size in the spongy and palisade mesophyll and lower cavity volume, other traits that have been found to arise in droughted plants, such as cell wall and cuticle thickness (Schimper 1903, Maximov 1929, Shields 1950), were not measured in this study. So it is possible that such changes did occur but were not detected. However, leaf thickness and LMA (or its inverse measure, specific leaf area) have been found to vary with water availability, and had marginally significant treatment effects (Table 4). Therefore, larger sample sizes might have revealed significant plot effects for these parameters.

### Summary

Changing climate is likely to exert selection pressure on the next generation of forest trees, but as the climate change is so rapid, the persistence and vigour of the current generation will be dependent to some extent on their ability to resist or acclimate to the new conditions (Nicotra et al. 2010). The taxa in this study responded to the imposed drought via changes in aspects of leaf anatomy that were not known to influence drought resistance, whilst exhibiting none of the expected changes. This might indicate that the experimental drought is not severe enough to influence leaf anatomy in the way expected, or that the traits typically associated with drought resistance are tightly constrained by other aspects of plant physiology. In the latter case, the restricted capacity for acclimation is suggestive of high sensitivity of this forest to climate change. The extent to which leaf anatomy determines the capacity of plants to cope with changes in water availability could have wide-reaching implications in understanding the drought sensitivity of plants; yet it is the subject of little research (Maximov 1929, Morton and Watson 1948, Shields 1950, Cutler et al. 1977) and has not been explored in an ecological context. Given that rainfall regimes are predicted to continue changing (Stocker 2014), and that the tropics in particular are likely to undergo more frequent and severe dry seasons (Christensen et al. 2013, Fu et al. 2013, Reichstein et al. 2013, Boisier et al. 2015), an improved understanding of these subjects could be invaluable to future estimates of forest vulnerability.

## Supplementary data

Supplementary data for this article are available at *Tree Physiology* Online.

Supplementary Data

## References

[tpw078C1] Aliniaeifard S, Matamoros PM, van Meeteren U (2014) Stomatal malfunctioning under low VPD conditions: induced by alterations in stomatal morphology and leaf anatomy or in the ABA signaling. Physiol Plant 152:688–699. 24773210 10.1111/ppl.12216

[tpw078C2] Allen CD, Breshears DD, McDowell NG (2015) On underestimation of global vulnerability to tree mortality and forest die-off from hotter drought in the Anthropocene. Ecosphere 6:1–55.

[tpw078C103] Allen CD, Macalady AK, Chenchouni H, Bachelet D, McDowell N, Vennetier M, Kitzberger T, Rigling A, Breshears DD, Hogg EH, Gonzalez P, Fensham R, Zhang Z, Castro J, Demidova N, Lim J, Allard G, Running SW, Semerci A, Cobb N (2010) A global overview of drought and heat-induced tree mortality reveals emerging climate change risks for forests. Forest Ecology and Management 259:660–684.

[tpw078C3] Anderegg LDL, Anderegg WRL, Berry JA (2013) Not all droughts are created equal: translating meteorological drought into woody plant mortality. Tree Physiol. 33:252–260 23880634 10.1093/treephys/tpt044

[tpw078C4] Anderegg WRL, Berry JA, Smith DD, Sperry JS, Anderegg LDL, Field CB (2012) The roles of hydraulic and carbon stress in a widespread climate-induced forest die-off. Proc Natl Acad Sci USA 109:233–237. 22167807 10.1073/pnas.1107891109 PMC3252909

[tpw078C5] Atkin OK, Tjoelker MG (2003) Thermal acclimation and the dynamic response of plant respiration to temperature. Trends Plant Sci 8:343–351. 12878019 10.1016/S1360-1385(03)00136-5

[tpw078C6] Bacelar EA, Correia CM, Moutinho-Pereira JM, Gonçalves BC, Lopes JI, Torres-Pereira JMG (2004) Sclerophylly and leaf anatomical traits of five field-grown olive cultivars growing under drought conditions. Tree Physiol 24:233–239. 14676039 10.1093/treephys/24.2.233

[tpw078C7] Bartlett MK, Scoffoni C, Sack L (2012) The determinants of leaf turgor loss point and prediction of drought tolerance of species and biomes: a global meta-analysis. Ecol Lett 15:393–405. 22435987 10.1111/j.1461-0248.2012.01751.x

[tpw078C101] Bartlett MK, Zhang Y, Kreidler N, Sun S, Ardy R, Cao K, Sack L (2014) Global analysis of plasticity in turgor loss point, a key drought tolerance trait. Ecology Letters 17:1580–1590. 10.1111/ele.12374 25327976

[tpw078C8] Binks O, Meir P, Rowland L et al (2016) Plasticity in leaf-level water relations of tropical rainforest trees in response to experimental drought. New Phytol 211:477–488 27001030 10.1111/nph.13927 PMC5071722

[tpw078C9] Boisier JP, Ciais P, Ducharne A, Guimberteau M (2015) Projected strengthening of Amazonian dry season by constrained climate model simulations. Nat Clim Change 5:656–661.

[tpw078C10] Bonan GB (2008) Forests and climate change: Forcings, feedbacks, and the climate benefits of forests. Science 320:1444–1449. 18556546 10.1126/science.1155121

[tpw078C11] Boughalleb F, Hajlaoui H (2011) Physiological and anatomical changes induced by drought in two olive cultivars (cv Zalmati and Chemlali). Acta Physiol Plant 33:53–65.

[tpw078C12] Bowman WD, Roberts SW (1985) Seasonal changes in tissue elasticity in chaparral shrubs. Physiol Plant 65:233–236.

[tpw078C83] Breshears DD, Cobb NS, Rich PM, Price KP, Allen CD, Balice RG, Romme WH, Kastens JH, Floyd ML, Belnap J, Anderson JJ, Myers OB, Meyer CW (2005) Regional vegetation die-off in response to global-change-type drought. Proc Natl Acad Sci USA 42:15144–15148. 10.1073/pnas.0505734102 PMC1250231 16217022

[tpw078C13] Brienen RJW, Phillips OL, Feldpausch TR et al (2015) Long-term decline of the Amazon carbon sink Nature 519:344–348. 25788097 10.1038/nature14283

[tpw078C14] Brodribb TJ, Feild TS, Sack L (2010) Viewing leaf structure and evolution from a hydraulic perspective. Funct Plant Biol 37:488–488.

[tpw078C15] Brodribb TJ, McAdam SAM (2015) Evolution in the smallest valves (stomata) guides even the biggest trees. Tree Physiol 35:451–452. 26041093 10.1093/treephys/tpv042

[tpw078C16] Buckley TN (2015) The contributions of apoplastic, symplastic and gas phase pathways for water transport outside the bundle sheath in leaves. Plant Cell Environ 38:7–22. 24836699 10.1111/pce.12372

[tpw078C17] Buckley TN, John GP, Scoffoni C, Sack L (2015) How does leaf anatomy influence water transport outside the xylem. Plant Physiol 168:1616–35. 26084922 10.1104/pp.15.00731 PMC4528767

[tpw078C18] Carins Murphy MR, Jordan GJ, Brodribb TJ (2014) Acclimation to humidity modifies the link between leaf size and the density of veins and stomata. Plant Cell Environ 37:124–131. 23682831 10.1111/pce.12136

[tpw078C19] Catoni R, Granata MU, Sartori F, Varone L, Gratani L (2015) *Corylus avellana* responsiveness to light variations: morphological, anatomical, and physiological leaf trait plasticity. Photosynthetica 53:35–46.

[tpw078C20] Chabot BF, Chabot JF (1977) Effects of light and temperature on leaf anatomy and photosynthesis in *Fragaria vesca*. Oecologia 26:363–377. 10.1007/BF00345535 28309501

[tpw078C21] Choat B, Jansen S, Brodribb TJ et al (2012) Global convergence in the vulnerability of forests to drought. Nature 491:752–755. 23172141 10.1038/nature11688

[tpw078C22] Christensen JH, Krishna Kumar K, Aldrian E et al (2013) Climate phenomena and their relevance for future regional climate change In: Stocker TF, Qin D, Plattner G-K et al. (eds) Climate change 2013: the physical science basis. Contribution of Working Group I to the Fifth Assessment Report of the Intergovernmental Panel on Climate Change. Cambridge University Press, Cambridge, UK, pp 1217–1308.

[tpw078C23] Cunningham SA, Summerhayes B, Westoby M (1999) Evolutionary divergences in leaf structure and chemistry, comparing rainfall and soil nutrient gradients. Ecol Monogr 69:569–588.

[tpw078C24] Cutler JM, Rains DW, Loomis RS (1977) Importance of cell-size in water relations of plants. Physiol Plant 40:255–260.

[tpw078C25] da Costa ACL, Galbraith D, Almeida S et al (2010) Effect of 7 yr of experimental drought on vegetation dynamics and biomass storage of an eastern Amazonian rainforest. New Phytol 187:579–591. 20553386 10.1111/j.1469-8137.2010.03309.x

[tpw078C26] Fisher RA, Williams M, da Costa AL, Malhi Y, da Costa RF, Almeida S, Meir P (2007) The response of an Eastern Amazonian rain forest to drought stress: results and modelling analyses from a throughfall exclusion experiment. Glob Chang Biol 13:2361–2378.

[tpw078C27] Fu R, Yin L, Li W et al (2013) Increased dry-season length over southern Amazonia in recent decades and its implication for future climate projection. Proc Natl Acad Sci USA 110:18110–18115. 24145443 10.1073/pnas.1302584110 PMC3831494

[tpw078C28] Galiano L, Martinez-Vilalta J, Sabate S, Lloret F (2012) Determinants of drought effects on crown condition and their relationship with depletion of carbon reserves in a Mediterranean holm oak forest. Tree Physiol 32:478–489. 22499595 10.1093/treephys/tps025

[tpw078C29] Geeske J, Aplet G, Vitousek PM (1994) Leaf morphology along environmental gradients in Hawaiian *Metrosideros polymorpha*. Biotropica 26:17–22.

[tpw078C30] Gianoli E (2001) Lack of differential plasticity to shading of internodes and petioles with growth habit in *Convolvulus arvensis* (Convolvulaceae). Int J Plant Sci 162:1247–1252.

[tpw078C31] Gleason SM, Westoby M, Jansen S et al (2015) Weak tradeoff between xylem safety and xylem-specific hydraulic efficiency across the world‘s woody plant species. New Phytol 209:123–136. 26378984 10.1111/nph.13646

[tpw078C32] Grace J, Mitchard E, Gloor E (2014) Perturbations in the carbon budget of the tropics. Glob Chang Biol 20:3238–3255. 24902948 10.1111/gcb.12600 PMC4261894

[tpw078C33] Hacke UG, Sperry JS, Pockman WT, Davis SD, McCulloh KA (2001) Trends in wood density and structure are linked to prevention of xylem implosion by negative pressure. Oecologia 126:457–461. 10.1007/s004420100628 28547229

[tpw078C34] Hajek P, Leuschner C, Hertel D, Delzon S, Schuldt B (2014) Trade-offs between xylem hydraulic properties, wood anatomy and yield in *Populus*. Tree Physiol 34:744–756. 25009155 10.1093/treephys/tpu048

[tpw078C35] Hanba YT, Kogami H, Terashima I (2002) The effect of growth irradiance on leaf anatomy and photosynthesis in *Acer* species differing in light demand. Plant Cell Environ 25:1021–1030.

[tpw078C36] Hartmann H, Ziegler W, Kolle O, Trumbore S (2013) Thirst beats hunger—declining hydration during drought prevents carbon starvation in Norway spruce saplings. New Phytol 200:340–349. 23692181 10.1111/nph.12331

[tpw078C37] Hsiao TC (1973) Plant responses to water stress. Annu Rev Plant Physiol 24:519–570.

[tpw078C38] Leuschner C (2002) Air humidity as an ecological factor for woodland herbs: leaf water status, nutrient uptake, leaf anatomy, and productivity of eight species grown at low or high vpd levels. Flora 197:262–274.

[tpw078C39] Lockhart JA (1965) An analysis of irreversible plant cell elongation. J Theor Biol 8:264–275. 5876240 10.1016/0022-5193(65)90077-9

[tpw078C40] Maximov NA (1929) The plant in relation to water. George Allen & Unwin, London, p 768.

[tpw078C41] McDowell NG (2011) Mechanisms linking drought, hydraulics, carbon metabolism, and vegetation mortality. Plant Physiol 155:1051–1059. 21239620 10.1104/pp.110.170704 PMC3046567

[tpw078C42] McDowell NG, Fisher RA, Xu C et al (2013) Evaluating theories of drought-induced vegetation mortality using a multimodel-experiment framework. New Phytol 200:304–321. 24004027 10.1111/nph.12465

[tpw078C43] McLean EH, Prober SM, Stock WD, Steane DA, Potts BM, Vaillancourt RE, Byrne M (2014) Plasticity of functional traits varies clinally along a rainfall gradient in *Eucalyptus tricarpa*. Plant Cell Environ 37:1440–1451. 24329726 10.1111/pce.12251

[tpw078C44] Mediavilla S, Escudero A, Heilmeier H (2001) Internal leaf anatomy and photosynthetic resource-use efficiency: interspecific and intraspecific comparisons. Tree Physiol 21:251–259. 11276419 10.1093/treephys/21.4.251

[tpw078C45] Meir P, Brando PM, Nepstad D et al (2009) The effects of drought on Amazonian rain forests In: Keller M, Bustamante M, Gash J, Dias PS (eds) Amazonia and global change. American Geophysical Union, Washington, D.C., USA, pp 429–449.

[tpw078C46] Meir P, Grace J (2005) The response to drought by tropical rain forest ecosystems In: Mahli Y, Phillips OL (eds) Tropical forests and global atmospheric change. Oxford University Press, Oxford, UK.

[tpw078C47] Meir P, Mencuccini M, Dewar RC (2015 *a*) Drought-related tree mortality: addressing the gaps in understanding and prediction. New Phytol 207:28–33. 25816852 10.1111/nph.13382

[tpw078C48] Meir P, Wood TE, Galbraith DR, Brando PM, Da Costa ACL, Rowland L, Ferreira LV (2015 *b*) Threshold responses to soil moisture deficit by trees and soil in tropical rain forests: insights from field experiments. Bioscience 65:882–892. 26955085 10.1093/biosci/biv107 PMC4777016

[tpw078C49] Metcalfe DB, Meir P, Aragão LEOC et al (2010) Shifts in plant respiration and carbon use efficiency at a large-scale drought experiment in the eastern Amazon. New Phytol 187:608–621. 20553394 10.1111/j.1469-8137.2010.03319.x

[tpw078C50] Morton AG, Watson DJ (1948) A physiological study of leaf growth. Ann Bot (Lond) 12:281–310.

[tpw078C51] Muller O, Cohu CM, Stewart JJ, Protheroe JA, Demmig-Adams B, Adams WW III (2014) Association between photosynthesis and contrasting features of minor veins in leaves of summer annuals loading phloem via symplastic versus apoplastic routes. Physiol Plant 152:174–183. 24450755 10.1111/ppl.12155

[tpw078C52] Nakagawa M, Tanaka K, Nakashizuka T et al (2000) Impact of severe drought associated with the 1997–1998 El Nino in a tropical forest in Sarawak. J Trop Ecol 16:355–367.

[tpw078C53] Nakagawa S, Schielzeth H (2013) A general and simple method for obtaining R2 from generalized linear mixed-effects models. Methods Ecol Evol 4:133–142.

[tpw078C54] Nicotra AB, Atkin OK, Bonser SP et al (2010) Plant phenotypic plasticity in a changing climate. Trends Plant Sci 15:684–692. 20970368 10.1016/j.tplants.2010.09.008

[tpw078C55] Niinemets Ü, Valladares F (2006) Tolerance to shade, drought, and waterlogging of temperate northern hemisphere trees and shrubs. Ecol Monogr 76:521–547.

[tpw078C56] Pan Y, Birdsey RA, Fang J et al (2011) A large and persistent carbon sink in the world's forests. Science 333:988–993. 21764754 10.1126/science.1201609

[tpw078C57] Phillips OL, Aragao LEOC, Lewis SL et al (2009) Drought sensitivity of the Amazon rainforest In: Science 323:1344–1347. 19265020 10.1126/science.1164033

[tpw078C58] R Core Team (2015) R: a language and environment for statistical computing. R Foundation for Statistical Computing, Vienna, Austria.

[tpw078C59] Reichstein M, Bahn M, Ciais P et al (2013) Climate extremes and the carbon cycle. Nature 500:287–295. 23955228 10.1038/nature12350

[tpw078C60] Revelle W (2015) Psych: Procedures for psychological, psychometric, and personality research. Northwestern University, Evanston, Illinois, USA.

[tpw078C61] Rockwell FE, Holbrook NM, Stroock AD (2014 *a*) The competition between liquid and vapor transport in transpiring leaves. Plant Physiol 164:1741–1758. 24572172 10.1104/pp.114.236323 PMC3982738

[tpw078C62] Rockwell FE, Michele Holbrook N, Stroock AD (2014 *b*) Leaf hydraulics I: Scaling transport properties from single cells to tissues. J Theor Biol 340:251–266. 24112968 10.1016/j.jtbi.2013.09.036

[tpw078C63] Roth I (1985) Stratification of tropical forests as seen in leaf structure. Tasks for vegetation science, Vol. 6. Dr W. Junk, The Hague, p. 464.

[tpw078C64] Rowland L, da Costa ACL, Galbraith DR et al (2015 *a*) Death from drought in tropical forests is triggered by hydraulics not carbon starvation. Nature 528:119–122. 26595275 10.1038/nature15539

[tpw078C65] Rowland L, Lobo-do-Vale RL, Christoffersen BO et al (2015 *b*) After more than a decade of soil moisture deficit, tropical rainforest trees maintain photosynthetic capacity, despite increased leaf respiration. Glob Chang Biol 21:4662–4672. 26179437 10.1111/gcb.13035 PMC4989466

[tpw078C66] Sack L, Pasquet-Kok J, PrometheusWiki contributors (2011) Leaf pressure-volume curve parameters. http://www.publish.csiro.au/prometheuswiki/tiki-pagehistory.php?page=Leaf pressure-volume curve parameters&preview=16 (5 July 2015, date last accessed)

[tpw078C67] Schimper AWF (1903) Plant-geography upon a physiological basis. Clarendon Press, Oxford, UK.

[tpw078C68] Schneider CA, Rasband WS, Eliceiri KW (2012) NIH Image to ImageJ: 25 years of image analysis Nat Methods 9:671–675. 22930834 10.1038/nmeth.2089 PMC5554542

[tpw078C69] Schoch PG, Zinsou C, Sibi M (1980) Dependence of the stomatal index on environmental-factors during stomatal differentiation in leaves of *Vigna sinensis* L. J Exp Bot 31:1211–1216.

[tpw078C70] Scoffoni C, Sack L, PrometheusWiki contributors (2010) Quantifying leaf vein traits. http://www.publish.csiro.au/prometheuswiki/tiki-pagehistory.php?page=Quantifying leaf vein traits&preview=15. (5 July 2015, date last accessed)

[tpw078C71] Sharkey TD, Bernacchi CJ, Farquhar GD, Singsaas EL (2007) Fitting photosynthetic carbon dioxide response curves for C-3 leaves. Plant Cell Environ 30:1035–1040. 17661745 10.1111/j.1365-3040.2007.01710.x

[tpw078C72] Shields LM (1950) Leaf xeromorphy as related to physiological and structural influences. Bot Rev 16:399–447.

[tpw078C73] Smith WK, Bell DT, Shepherd KA (1998) Associations between leaf structure, orientation, and sunlight exposure in five Western Australian communities. Am J Bot 85:56–63. 21684880

[tpw078C74] Stocker TF (2014) Climate change 2013: the physical science basis: working group I contribution to the fifth assessment report of the intergovernmental panel on climate change. Cambridge University Press, New York, NY, USA.

[tpw078C75] Tyree MT, Hammel HT (1972) Measurement of turgor pressure and water relations of plants by pressure-bomb technique. J Exp Bot 23:267–282.

[tpw078C76] Valladares F, Gianoli E, Gómez JM (2007) Ecological limits to plant phenotypic plasticity. New Phytol 176:749–763. 17997761 10.1111/j.1469-8137.2007.02275.x

[tpw078C77] Van Kleunen M, Fischer M (2005) Constraints on the evolution of adaptive phenotypic plasticity in plants. New Phytol 166:49–60. 15760350 10.1111/j.1469-8137.2004.01296.x

[tpw078C78] Venables WN (2002) Modern applied statistics with S, 4th edn Springer, New York.

[tpw078C79] Walls RL (2011) Angiosperm leaf vein patterns are linked to leaf functions in a global-scale data set. Am J Bot 98:244–253. 21613113 10.3732/ajb.1000154

[tpw078C80] Warren CR, Tausz M, Adams MA (2005) Does rainfall explain variation in leaf morphology and physiology among populations of red ironbark (*Eucalyptus sideroxylon* subsp *tricarpa*) grown in a common garden. Tree Physiol 25:1369–1378. 16105804 10.1093/treephys/25.11.1369

[tpw078C81] Wylie RB (1943) The role of the epidermis in foliar organization and its relations to the minor venation. Am J Bot 30:273–280.

[tpw078C82] Zhang S-B, Sun M, Cao K-F, Hu H, Zhang J-L (2014) Leaf photosynthetic rate of tropical ferns is evolutionarily linked to water transport capacity. PLoS One 9:1–10. 10.1371/journal.pone.0084682 PMC3886989 24416265

