# Supplementary material for: Limited acclimation in leaf anatomy to experimental drought in tropical rainforest trees
Source: Tree Physiol. 2016 Dec 19;36(12):1550–61. doi: 10.1093/treephys/tpw078 (PMC5165703; doi:10.1093/treephys/tpw078)
Supplement: Supplementary Data [file supp_36_12_1550__index.html]

Limited acclimation in leaf anatomy to experimental drought in tropical rainforest trees — Limited acclimation in leaf anatomy to experimental drought in tropical rainforest trees — Supplementary Data 

# Limited acclimation in leaf anatomy to experimental drought in tropical rainforest trees

## Supplementary Data

Supplementary Data

- Supplementary Data - docx file
