# Supplementary material for: Limited acclimation in leaf anatomy to experimental drought in tropical rainforest trees
Source: Tree Physiol. 2016 Dec 19;36(12):1550–61. doi: 10.1093/treephys/tpw078 (PMC5165703; doi:10.1093/treephys/tpw078)
Supplement: Supplementary Data [file supp_tpw078_Binks_SI.docx]

# Limited acclimation in leaf anatomy to experimental drought in tropical rainforest trees

## Supplementary Information

Table S1. The number of leaves used in the analysis for each variable. The minimum and mean number of leaves is shown ‘per genus’, ‘per genus per plot’, and ‘per drought sensitivity status per plot’. The genus represented by the minimum number of leaves is shown where Esch is *Eschweilera*, Prot is *Protium*, Pout is *Pouteria*, and Man is *Manilkara*. In the drought sensitivity per plot category, the minimally represented group is indicated where the control plot is A, the drought plot is B, drought resistant species are R and drought sensitive species are S. Thus, AS represents the drought sensitive species in the control plot.

|  |  | genus | | genus per plot | | drought sensitivity  per plot | |
| --- | --- | --- | --- | --- | --- | --- | --- |
| Variable | total | min | mean | min | mean | min | mean |
| Leaf area | 400 | 57, Esch | 67 | 23, Esch | 33 | 93, AS | 100 |
| Leaf thickness | 68 | 6, Prot | 11 | 2, Prot | 6 | 13, AR | 17 |
| Leaf mass per area | 392 | 56, Esch | 65 | 23, Esch | 33 | 93, AS | 98 |
| Vein density | 60 | 7, Pout (0, Man) | 12 | 2, Lic | 6 | 10, AS | 15 |
| Stomatal density | 112 | 4, Lic | 19 | 0, Lic | 10 | 18, BR | 28 |
| Spongy mesophyll thickness | 72 | 6, Prot | 12 | 2, Prot | 6 | 14, AR | 18 |
| Palisade mesophyll thickness | 76 | 6, Prot | 13 | 2, Prot | 6 | 15, AR | 19 |
| Adaxial epidermis thickness | 72 | 6, Prot | 12 | 2, Prot | 6 | 14, AR | 18 |
| Abaxial epidermis thickness | 66 | 6, Prot, Man | 11 | 2, Prot, Man | 6 | 13, AR | 17 |
| Internal cavity volume | 56 | 5, Pout | 9 | 2, Pout | 5 | 13, BR | 13 |
| Proportional SM thickness | 61 | 4, Man | 10 | 1, Man | 5 | 12, AR | 15 |
| Proportional Pal thickness | 61 | 4, Man | 10 | 1, Man | 5 | 12, AR | 15 |
| Proportional Ab thickness | 61 | 4, Man | 10 | 1, Man | 5 | 12, AR | 15 |
| Proportional Ad thickness | 61 | 4, Man | 10 | 1, Man | 5 | 12, AR | 15 |
| Proportional CV thickness | 29 | 3, Prot | 5 | 1, Prot | 2 | 7, AR, BR, BS | 7 |
| SM cell volume | 55 | 6, Prot, Man | 9 | 2, Prot | 5 | 12, AR, BR | 14 |
| Pal cell volume | 57 | 6, Prot, Man | 10 | 2, Prot | 5 | 12, BR | 14 |

Table S2. Pearson correlation coefficients (r) above the diagonal, and probability values below the diagonal, derived from Pearson correlation analyses. No correction for multiple tests applied. P values < 0.05 and their associated r statistic are highlighted in bold and variables are arranged from the highest number of correlations in the top left, to the lowest in the bottom right. Symbols as defined in Table 1 with additional variables: absolute and proportional palisade mesophyll thickness (Pal and Pal_prop_, respectively), osmotic potential at full turgor ($\Psi_{\pi}^{o}$), turgor loss point ($\Psi_{\pi}^{tlp}$)_,_ saturated water content (SWC), elastic modulus (ε), hydraulic capacitance (C), relative water content at $\Psi_{\pi}^{tlp}$ (RWC^tlp^), predawn water potential $\Psi_{PD}$, electron transport driving regeneration of RuBP (J_max_), the maximum rate of rubisco carboxylation (V_cmax_), and dark respiration (R_dark_).

|  | Ad_prop_ | SM | T | Ψ_π_^O^ | Ψ_π_^tlp^ | Ad | SM_prop_ | Pal | SWC |
| --- | --- | --- | --- | --- | --- | --- | --- | --- | --- |
| Ad_prop_ |  | **-0.73** | **-0.54** | **-0.59** | **-0.59** | **0.69** | **-0.67** | -0.18 | **-0.4** |
| SM | **0** |  | **0.85** | **0.43** | **0.43** | -0.17 | **0.69** | **0.41** | **0.53** |
| T | **0** | **0** |  | 0.3 | 0.29 | 0.17 | 0.28 | **0.67** | **0.52** |
| Ψ_π_^O^ | **0** | **0.01** | 0.08 |  | **0.97** | **-0.5** | **0.39** | 0.09 | **0.46** |
| Ψ_π_^tlp^ | **0** | **0.01** | 0.09 | **0** |  | **-0.52** | **0.39** | 0.03 | **0.5** |
| Ad | **0** | 0.33 | 0.32 | **0** | **0** |  | **-0.5** | 0.31 | -0.09 |
| SM_prop_ | **0** | **0** | 0.1 | **0.02** | **0.02** | **0** |  | -0.26 | 0.27 |
| Pal | 0.31 | **0.01** | **0** | 0.58 | 0.88 | 0.06 | 0.13 |  | 0.07 |
| SWC | **0.02** | **0** | **0** | **0** | **0** | 0.59 | 0.11 | 0.7 |  |
| Ab | 0.73 | **0.01** | **0** | 0.38 | 0.33 | **0.01** | 0.78 | **0** | 0.06 |
| Pal_prop_ | **0** | **0** | **0.03** | 0.06 | **0.01** | 0.22 | **0** | **0.01** | **0** |
| RWC^tlp^ | **0.02** | **0** | **0** | **0** | **0** | 0.88 | 0.26 | 0.06 | **0.02** |
| SM_cell_volume_ | **0** | **0** | **0.01** | **0.02** | **0.02** | 0.18 | **0** | 0.75 | 0.05 |
| VD | **0** | **0** | **0.01** | 0.38 | 0.27 | 0.48 | **0.04** | 0.43 | **0** |
| ε | **0.03** | 0.49 | 0.34 | **0** | **0** | **0** | **0.01** | 0.26 | 0.2 |
| C | **0.03** | **0.03** | 0.18 | **0** | **0** | 0.12 | 0.2 | 0.33 | **0** |
| Ab_prop_ | **0** | **0.01** | 0.2 | **0** | **0** | **0.01** | **0** | 0.67 | 0.21 |
| J_max_ | **0** | **0.01** | **0** | 0.26 | 0.29 | 0.35 | 0.68 | **0.01** | 0.07 |
| SD | 0.13 | **0.01** | **0** | 0.14 | 0.16 | 0.11 | 0.71 | **0.01** | **0.04** |
| V_cmax_ | **0** | **0.01** | **0** | 0.5 | 0.49 | 0.11 | 0.4 | **0.01** | 0.25 |
| Pal_cell_volume_ | **0.01** | 0.09 | 0.08 | **0.03** | 0.17 | 0.05 | 0.28 | **0.02** | 0.64 |
| LMA | 0.73 | 0.07 | **0** | 0.35 | 0.34 | **0** | 0.23 | **0** | 0.74 |
| PD | **0.01** | 0.1 | 0.35 | **0.02** | 0.09 | **0.01** | 0.06 | 0.21 | 0.65 |
| CV | 0.75 | 0.37 | **0.01** | 0.99 | 0.69 | **0.04** | 0.19 | 0.13 | 0.08 |
| R_dark_ | 0.27 | 0.12 | 0.08 | 0.42 | 0.29 | 0.62 | 0.77 | **0.01** | 0.6 |
| CV_prop_ | 0.05 | 0.3 | 0.65 | 0.44 | 0.69 | **0.01** | **0.02** | 1 | 0.6 |
| Area | 0.41 | 0.71 | 0.3 | 0.94 | 0.9 | 0.87 | 0.17 | **0.04** | 0.98 |
|  |  |  |  |  |  |  |  |  |  |
|  |  |  |  |  |  |  |  |  |  |

|  | Ab | Pal_prop_ | RWC^tlp^ | SM_cell_volume_ | VD | ε | C | Ab_prop_ | J_max_ |
| --- | --- | --- | --- | --- | --- | --- | --- | --- | --- |
| Ad_prop_ | -0.06 | **0.47** | **-0.41** | **-0.49** | **0.57** | **0.38** | **-0.38** | **0.55** | **-0.48** |
| SM | **0.43** | **-0.54** | **0.52** | **0.5** | **-0.64** | -0.12 | **0.36** | **-0.46** | **0.4** |
| T | **0.72** | **-0.38** | **0.68** | **0.45** | **-0.52** | 0.16 | 0.23 | -0.23 | **0.48** |
| Ψ_π_^O^ | -0.15 | -0.33 | **0.43** | **0.41** | -0.17 | **-0.6** | **0.57** | **-0.61** | 0.18 |
| Ψ_π_^tlp^ | -0.17 | **-0.44** | **0.48** | **0.41** | -0.21 | **-0.5** | **0.57** | **-0.61** | 0.17 |
| Ad | **0.44** | 0.21 | 0.03 | -0.24 | 0.15 | **0.63** | -0.27 | **0.42** | -0.16 |
| SM_prop_ | -0.05 | **-0.66** | 0.19 | **0.49** | **-0.41** | **-0.41** | 0.22 | **-0.48** | 0.07 |
| Pal | **0.51** | **0.42** | 0.32 | 0.06 | -0.17 | 0.19 | 0.17 | -0.08 | **0.4** |
| SWC | 0.32 | **-0.5** | **0.35** | 0.35 | **-0.62** | -0.2 | **0.44** | -0.22 | 0.28 |
| Ab |  | -0.19 | **0.37** | 0.11 | -0.37 | **0.43** | -0.02 | **0.52** | 0.29 |
| Pal_prop_ | 0.29 |  | **-0.43** | **-0.4** | **0.48** | 0.06 | -0.07 | 0.2 | -0.18 |
| RWC^tlp^ | **0.03** | **0.01** |  | **0.5** | -0.34 | 0.27 | -0.12 | -0.29 | 0.24 |
| SM_cell_volume_ | 0.55 | **0.02** | **0** |  | **-0.66** | -0.24 | 0.1 | **-0.36** | 0.1 |
| VD | 0.07 | **0.02** | 0.07 | **0** |  | -0.09 | -0.31 | 0.1 | **-0.44** |
| ε | **0.01** | 0.72 | 0.08 | 0.18 | 0.66 |  | **-0.56** | **0.43** | -0.01 |
| C | 0.9 | 0.71 | 0.46 | 0.57 | 0.11 | **0** |  | -0.34 | 0.23 |
| Ab_prop_ | **0** | 0.25 | 0.1 | **0.04** | 0.65 | **0.01** | 0.05 |  | -0.16 |
| J_max_ | 0.08 | 0.32 | 0.13 | 0.58 | **0.02** | 0.96 | 0.14 | 0.38 |  |
| SD | **0.02** | 0.39 | 0.23 | 0.29 | **0.01** | 0.9 | **0.02** | 0.75 | 0.06 |
| V_cmax_ | 0.21 | 0.37 | 0.25 | 0.59 | **0.03** | 0.85 | 0.49 | 0.21 | **0** |
| Pal_cell_volume_ | 0.75 | 0.28 | **0.04** | 0.32 | 0.78 | 0.27 | 0.55 | 0.12 | **0.04** |
| LMA | **0.01** | 0.4 | 0.26 | 0.99 | 0.45 | 0.1 | 0.46 | 0.8 | 0.09 |
| PD | 0.74 | 0.38 | 0.78 | 0.51 | 0.76 | **0.02** | **0.01** | 0.14 | 0.14 |
| CV | **0.01** | 0.22 | 0.07 | 0.57 | 0.43 | 0.12 | 0.31 | 0.73 | **0.03** |
| R_dark_ | 0.26 | 0.4 | 0.74 | 0.52 | 0.21 | 0.73 | 0.78 | 0.83 | **0** |
| CV_prop_ | 0.23 | 0.8 | 0.45 | 0.15 | 0.89 | 0.11 | 0.78 | 0.35 | 0.52 |
| Area | 0.51 | 0.26 | 0.45 | 0.51 | 0.92 | 0.94 | **0.02** | 0.86 | 0.07 |

|  | SD | V_cmax_ | Pal_cell_volume_ | LMA | PD | CV | R_dark_ | CV_prop_ | Area |
| --- | --- | --- | --- | --- | --- | --- | --- | --- | --- |
| Ad_prop_ | 0.3 | **-0.55** | **-0.47** | 0.07 | **0.42** | 0.06 | 0.19 | 0.38 | 0.16 |
| SM | **-0.48** | **0.45** | 0.3 | 0.33 | -0.27 | 0.17 | -0.26 | -0.2 | -0.07 |
| T | **-0.56** | **0.46** | 0.31 | **0.56** | -0.16 | **0.47** | -0.3 | 0.09 | -0.19 |
| Ψ_π_^O^ | -0.28 | 0.11 | **0.37** | -0.16 | **-0.36** | 0 | 0.13 | -0.15 | 0.01 |
| Ψ_π_^tlp^ | -0.27 | 0.11 | 0.25 | -0.17 | -0.27 | 0.07 | 0.17 | -0.08 | 0.02 |
| Ad | -0.32 | -0.27 | -0.34 | **0.57** | **0.43** | **0.39** | 0.08 | **0.49** | 0.03 |
| SM_prop_ | -0.08 | 0.14 | 0.19 | -0.22 | -0.31 | -0.25 | -0.05 | **-0.43** | 0.25 |
| Pal | **-0.49** | **0.43** | **0.41** | **0.78** | -0.21 | 0.29 | **-0.42** | 0 | **-0.37** |
| SWC | **-0.38** | 0.18 | 0.09 | -0.06 | -0.07 | 0.32 | -0.08 | 0.1 | 0 |
| Ab | **-0.44** | 0.21 | 0.06 | **0.48** | 0.06 | **0.47** | -0.19 | 0.24 | -0.13 |
| Pal_prop_ | 0.18 | -0.16 | 0.2 | 0.17 | -0.15 | -0.25 | -0.15 | -0.05 | -0.22 |
| RWC^tlp^ | -0.23 | 0.18 | **0.37** | 0.2 | -0.04 | 0.33 | 0.05 | 0.15 | 0.13 |
| SM_cell_volume_ | -0.22 | 0.1 | 0.18 | 0 | -0.12 | -0.12 | -0.12 | -0.29 | 0.13 |
| VD | **0.56** | **-0.4** | -0.06 | -0.16 | -0.06 | -0.17 | 0.24 | 0.03 | -0.02 |
| ε | -0.02 | 0.03 | -0.2 | 0.29 | **0.37** | 0.28 | 0.06 | 0.3 | 0.01 |
| C | **-0.44** | 0.11 | 0.11 | 0.13 | **-0.4** | 0.19 | -0.04 | 0.05 | **-0.38** |
| Ab_prop_ | 0.07 | -0.22 | -0.28 | 0.05 | 0.26 | 0.07 | 0.04 | 0.19 | 0.04 |
| J_max_ | -0.35 | 0.86 | **0.36** | **0.29** | -0.23 | **0.38** | **-0.51** | 0.12 | -0.31 |
| SD |  | -0.28 | -0.05 | **-0.43** | 0.2 | -0.34 | -0.07 | -0.19 | -0.02 |
| V_cmax_ | 0.14 |  | 0.28 | 0.27 | -0.12 | 0.28 | **-0.5** | -0.02 | -0.17 |
| Pal_cell_volume_ | 0.82 | 0.11 |  | 0 | **-0.71** | -0.18 | **-0.38** | -0.34 | -0.06 |
| LMA | **0.04** | 0.12 | 1 |  | -0.09 | **0.47** | -0.3 | 0.28 | **-0.36** |
| PD | 0.3 | 0.46 | **0** | 0.61 |  | 0.25 | 0.23 | **0.39** | 0.22 |
| CV | 0.12 | 0.13 | 0.39 | **0.01** | 0.17 |  | -0.13 | **0.89** | -0.22 |
| R_dark_ | 0.7 | **0** | **0.03** | 0.08 | 0.15 | 0.47 |  | 0.17 | 0.29 |
| CV_prop_ | 0.43 | 0.9 | 0.1 | 0.14 | **0.04** | **0** | 0.39 |  | -0.11 |
| Area | 0.92 | 0.32 | 0.76 | **0.03** | 0.21 | 0.24 | 0.09 | 0.58 |  |
